# Alternatives for the Detection and Diagnosis of Osteoarticular Infections: An Exploratory Review

**DOI:** 10.7759/cureus.63743

**Published:** 2024-07-03

**Authors:** Esteban Zavaleta-Monestel, Mariela Alpizar-Rojas, Jonathan García-Montero, Alexa León-Obando, Sebastián Arguedas-Chacón, Ricardo Quesada-Villaseñor

**Affiliations:** 1 Pharmacy, Hospital Clínica Bíblica, San José, CRI; 2 Pharmacy, Universidad de Iberoámerica, San José, CRI; 3 Pharmacy and Nuclear Medicine, Hospital Clínica Bíblica, San José, CRI; 4 Pharmacy and Clinical Research, Hospital Clínica Bíblica, San José, CRI; 5 Medical Research, Hospital Clínica Bíblica, San José, CRI

**Keywords:** infection, osteoarticular disease, 99mtc-ciprofloxacin, technetium-99m, gallium-67, osteomyelitis

## Abstract

The precise diagnosis of osteomyelitis, a bone infection, remains a significant challenge for healthcare professionals. This difficulty stems from the highly variable nature of its clinical presentation and disease course. Patients can exhibit a wide range of symptoms, making it easy to misdiagnose the condition. In turn, inaccurate diagnoses lead to inappropriate treatment regimens, potentially hindering a patient’s recovery and causing unnecessary complications. Nuclear medicine offers a ray of hope in this fight against diagnostic ambiguity. It provides valuable tools, such as radiopharmaceutical imaging, that can significantly improve the accuracy of osteomyelitis diagnosis. However, limitations exist. This article explores the need for alternative diagnostic approaches within the specific context of Costa Rica. This exploration is particularly relevant due to the current regional shortage of gallium-67 (⁶⁷Ga), a radiopharmaceutical commonly used in osteomyelitis diagnosis. The article delves into the nature, function, and limitations of various nuclear medicine techniques, encompassing both independent radiopharmaceuticals like ⁶⁷Ga and those conjugated with specific targeting molecules to pinpoint areas of infection within the body. Given the scarcity of ⁶⁷Ga in Costa Rica, it becomes crucial to explore and implement viable alternative diagnostic techniques within the healthcare system. This article emphasizes the need for further investigation into these alternatives, with the goal of improving diagnostic accuracy and ensuring optimal patient care. By implementing these alternatives, healthcare professionals in Costa Rica can effectively combat the challenges posed by osteomyelitis and pave the way for better patient outcomes.

## Introduction and background

Nuclear medicine is the branch of health sciences that utilizes radioactive isotopes in small quantities to prevent, diagnose, define therapies, treat diseases, and even play a significant role in medical research. This field collaborates closely with radiology, neurology, and oncology specialties [1]. It is primarily based on administering a small dose of the radioactive substance, known as a radiopharmaceutical. The selection of the radiopharmaceutical varies depending on the purpose of the scan, which can be used to examine the structure or function of an organ. The images generated in nuclear medicine assist in diagnosing diseases such as tumors, and infections in various tissues, including bone, and other cardiovascular conditions [1].

Some of the diagnoses offered by nuclear medicine can be described, including analyzing kidney function, obtaining images of blood circulation and heart function, evaluating bone fractures, infections, arthritis, or tumors, determining the presence of any cancer spreading in the body, conducting heart studies, and verifying the effectiveness of applied treatments [1,2]. Nuclear medicine utilizes radiopharmaceuticals, also known as tracers, for diagnostic purposes. These tracers are introduced into the patient’s body, and specialized imaging cameras can then detect their emissions from outside the body, allowing for the tracking of the radiopharmaceutical distribution within the organism [3].

Nuclear medicine is a versatile tool with numerous clinical applications. This review focuses on its role in diagnosing osteomyelitis, a bone pathology with a high prevalence of comorbidities. Osteomyelitis can significantly impact a patient’s health, and its diagnosis often necessitates a comprehensive approach that incorporates various diagnostic studies [4]. In such cases, nuclear medicine procedures become crucial for obtaining a more precise assessment of the disease [5].

Osteomyelitis is an infection caused by the inoculation of microbial organisms. It is characterized by a low incidence; for example, it has been observed in 50% of the pediatric population under five years old and at least 25% in those under two years old, with the remaining 50% affecting the adult population [6]. It is generally a pathology influenced by various risk factors that increase the likelihood of its development, such as diabetes mellitus, pressure ulcers, surgery, trauma, and intravenous drug use [4]. There are multiple classifications of osteomyelitis, with one of the most common being the Waldvogel classification. This classification divides osteomyelitis into acute or hematogenous osteomyelitis (spread to soft tissues), chronic osteomyelitis, osteomyelitis secondary to an adjacent focus due to surgical intervention (e.g., as a result of a process involving the use of prosthetics or trauma), and vascular insufficiency-related osteomyelitis (generalized and nongeneralized), which commonly occurs in patients with type 2 diabetes mellitus [4,6].

The role of nuclear medicine in the differential diagnosis of osteoarticular pathologies is crucial. Through bone scintigraphy, significant information can be obtained due to the radiopharmaceutical’s affinity for bone, maintaining its osteoblastic capacity. This study aids in the detection of osteomyelitis 10-14 days before it becomes visible on a plain X-ray [1,7]. In nuclear medicine imaging for diagnosing infections, radiotracers play a critical role. These radiopharmaceuticals can be used independently, like gallium-67 (⁶⁷Ga), or conjugated (tagged) to other molecules or cells with a specific affinity for infected areas. Examples of such radiotracers include technetium-99m (Tc-99m), indium-111 (In-111), and others [3,8]. For gamma camera imaging of osteomyelitis, two commonly used radiotracers are ⁶⁷Ga and labeled leukocytes [3].

The main objective of this study is to evaluate alternative diagnostic approaches in Costa Rica for detecting and diagnosing osteoarticular infections, such as osteomyelitis, particularly those that can be used in situations where gallium-67 is not readily available. Additionally, it aims to offer healthcare professionals involved in diagnosis additional options by leveraging existing resources, such as the use of technetium-99m and ciprofloxacin.

## Review

Discussion

Osteomyelitis

Osteomyelitis refers to an infection in the bone that generates an inflammatory process and an increase in intraosseous pressure, specifically in the marrow, cortex, and periosteum [6,7,9]. It is characterized by progressive growth and, additionally, by causing ischemic destruction of the skeletal tissue [6]. The microorganisms that frequently cause osteomyelitis are first, with the highest percentage,* Staphylococcus *epidermis with a 30% probability, followed by *Staphylococcus aureus* with a 29% probability, and third, with the highest percentage, methicillin-resistant Staphylococcus epidermidis with a 13% probability [10].

The pathophysiology of this infection can manifest through different mechanisms, such as hematogenous spread, through an exposed fracture, and as complications of other upper respiratory tract infections, including otitis and sinusitis, among others [11]. Once the microorganism enters and reaches the bone tissue, an inflammatory process is triggered, which can be explained as follows: first, the pathogen, through proteins called adhesins, attaches to collagen and cartilage, leading to a decrease in blood flow and occlusion of the capillaries within the bone tissue [4,7]. This process can cause necrosis, leading the infectious agent to locally disseminate, forming abscesses that spontaneously drain [7].

Within this process, osteoblasts, cells responsible for bone formation, various cytokines and interleukins such as IL-1, IL-6, IL-11, tumor necrosis factor, and the presence of osteoclasts initiate an inflammatory process. It is important to note that macrophages release proteolytic enzymes along with free radicals to degrade the surrounding bone tissue. The result is a generalized inflammatory process where tissue remodeling is delayed due to the infection itself [4,7].

There are different classifications of osteomyelitis, and with them, the intensity of symptoms varies. The Waldvogel classification divides it based on the duration of the disease and the route or mechanism of infection. The first is subdivided into two types: the acute phase, characterized by mild inflammation in the bone, and the release of various inflammatory mediators by the microorganism. In this stage, the main symptoms are fever and redness in the affected area. On the other hand, in the chronic state, there is the presence of necrotic bone, which can lead to recurrent episodes of infection. Prominent symptoms in this phase include the formation of fistulas and the creation of purulent bone tissue [4,11,12]. The second subdivision includes hematogenous osteomyelitis, commonly caused by the spread of microorganisms transported in the bloodstream. It is more frequent in the pediatric population, and when it occurs in adults, it is associated with predisposing diseases. Last, there is osteomyelitis associated with a contiguous focus of infection, which can appear in the presence of surgery or trauma to the tissue [11,12].

Currently, one of the most significant challenges in the diagnosis of osteomyelitis lies in the fact that this pathology exhibits highly variable symptoms across different age groups and can be unpredictable [6]. The diagnostic process for this disease begins with the patient’s medical history, laboratory tests, and imaging techniques that aid in generating a more precise diagnosis of osteomyelitis. It is important to note that, except for radiology, more specialized imaging is only conducted in cases where the diagnosis is not clear [6,9].

Nuclear medicine studies in such cases involve scintigraphy, as they provide images of the musculoskeletal system. By utilizing radiopharmaceuticals, areas where bone tissue exhibits increased osteoblastic activity can be observed [9]. Within the realm of differential diagnoses, conditions that may lead to a misinterpretation of osteomyelitis include certain cardiac conditions, recent trauma, rheumatoid arthritis, septic arthritis, and even joint and epiphyseal tuberculosis [12].

Diagnostic Alternatives

Gallium-67: Gallium-67 (⁶⁷Ga) is a radioisotope with the ability to emit gamma radiation at different energies, possessing a physical half-life of 77.8 hours. Administered intravenously, ⁶⁷Ga can bind serum proteins, specifically transferrin and haptoglobin, providing it with the ability to detect inflammatory processes [13]. Its source is obtained through a cyclotron, an apparatus in which charged particles are accelerated in a vacuum by the action of an electromagnetic field, following a circular trajectory. In each revolution, the accelerated particles gain energy sufficient to be released in the form of a beam onto an appropriate target [14].

Based on our hospital’s experience, it is recommended to undergo a three-phase bone scintigraphy with recent 99mTc-MDP/HDP, ideally performed within a month and a half. It is important to note that, during both studies, after the administration of the radiopharmaceutical, the patient can resume their normal activities and then return to the nuclear medicine service for image acquisition in the gamma camera. Additionally, the significance of ensuring an empty bladder during the procedure is emphasized, as the presence of the radiopharmaceutical in the bladder was previously observed, which could interfere with the medical interpretation of the study. Studies conducted with gallium-67 have had to be halted due to its global shortage. This is attributed to a recent communication from China, indicating restrictions on the distribution of radioactive material to certain countries. Such restrictions have resulted in negative implications for the diagnostic approach to detecting osteoarticular infections in patients [15].

In Costa Rica, this radiopharmaceutical is obtained through distributors who import it, as there is no internal production. However, since 2019, there has been limited supply from these entities, who report that the number of producers of this radioactive isotope is decreasing. In the Costa Rican market, one of the platforms most utilized is Bioplus Care, a major supplier of radiopharmaceuticals. Through its partnership with the French company Curium™, this company enabled the Ministry of Health to have gallium-67 as a registered product [16]. However, the temporary suspension of its production by Curium™ has led to a shortage of supplies in the western region.

Considering the foregoing, the limitations of current diagnostic methods and the scarcity of gallium-67 in the western region necessitate the urgent development of alternative approaches for diagnosing osteoarticular infections, particularly osteomyelitis.

Indium-111-labeled leukocytes: Indium-111-labeled leukocytes are a type of radiopharmaceutical used for diagnostic imaging. They emit gamma radiation and, through internal conversion, can also emit X-rays. Administered intravenously, these leukocytes contain a small amount of radioactive activity that allows for the visualization of specific organ functions. Indium-111 (In-111), the radioisotope used to label the leukocytes, possesses a physical half-life of approximately 67.2 hours [17].

To perform labeling with leukocytes, it is crucial to have the appropriate equipment and consider the necessary precautions for handling biological fluids with a high potential for contagion [18,19]. First, a blood sample (in milliliters) must be extracted from the patient and mixed with an anticoagulant solution. At least 2 × 108 leukocytes are needed. Next, it is important to separate the plasma, as it contains proteins that could degrade the In-111 complex, primarily due to the metal-chelating activity. Subsequently, the sample is centrifuged at 2,000 g using a Falcon tube for 10 minutes, and then the supernatant is extracted [18].

Another relevant aspect of the labeling process involves adding 20 MBq of In-111 to the solution containing leukocytes in a buffered pharmaceutical solution. It is left to incubate for approximately 10 minutes at room temperature. Finally, a saline solution is added, and the mixture is centrifuged again at 150 g for five minutes to extract the supernatant containing excess In-111 [19,20]. Indium-111 labeling offers greater stability compared to Technetium-99m (99mTc). This advantage arises because 99mTc elutes from leukocytes at a faster rate than 111In [3].

A significant limitation is the current lack of a supplier authorized to import In-111 into Costa Rica. Consequently, there is no regulatory approval for its use within the country. Furthermore, Costa Rica presently lacks the domestic capability to produce In-111.

Technetium-99m labeled leukocytes: The diagnostic technique of leukocytes labeled with Tc-99m functions similarly to the previous ones; for example, three-phase scintigraphy using 99mTc-MDP is required before this study [17]. Tc-99m has a half-life of approximately six hours. For this study, a minimum activity of 8.2 + 2.2 mCi is needed. Regarding leukocyte preparation, the process follows the guidelines outlined by the European Society of Nuclear Medicine. Once the radiopharmaceutical is prepared, it is administered to the patient for image acquisition, typically ranging from 30 minutes to 24 hours post-administration [20].

Among the limitations of this study in Costa Rica is the absence of a Ministry of Health registration authorizing its use. Additionally, proper handling requires an appropriate space for managing biological material.

99MTc-ciprofloxacin: Technetium-99m is a radioactive isotope with the ability to emit gamma rays that are captured by the gamma camera to generate images of the interior of the body [21]. As mentioned earlier, Tc-99m has a half-life of approximately six hours, providing sufficient time for diagnostic studies without subjecting the patient to prolonged radiation exposure [22]. Tc-99m, on its own, exhibits affinity only for the thyroid gland. Therefore, it is combined with a molecule that has an affinity for the specific organ under-diagnosis. The production of this radioactive isotope involves irradiating uranium with neutron beams for about a week in a nuclear reactor. This process chemically separates molybdenum from uranium, yielding high-purity Mo-99. Subsequently, Mo-99 is deposited into the Mo-99/Tc-99m generator and sent to nuclear medicine services [23]. Numerous studies conducted over time have determined a sensitivity of 97.6% and a specificity of 100%, with positive and negative predictive values of 100% and 87.5%, respectively. These values demonstrate the effectiveness of Tc-99m in diagnostic imaging [24,25].

The broad-spectrum antibacterial activity and widespread availability of ciprofloxacin motivated its radiolabeling 99mTc for evaluation as a potential imaging agent for deep-seated infections [26]. Diagnosing these infections often proves challenging due to conflicting information obtained with conventional imaging methods. 99mTc-ciprofloxacin, however, has shown promising results in accurately diagnosing such infections [27,28].

Additionally, there are studies utilizing a freeze-dried kit containing 99mTc-ciprofloxacin obtained from Uruguay. The preparation involves adding 1-3 milliliters of a solution containing 99mTc-pertechnetate to the lyophilized kit containing 4 mg of ciprofloxacin, with the solution having a maximum activity of 50 mCi. After introducing technetium-99m, the radiopharmaceutical is agitated for approximately two minutes, left to rest at room temperature, and protected from light due to photosensitivity [29]. Following intravenous administration, imaging begins approximately 60 minutes after the injection, with additional images acquired at four hours and up to 24 hours post-administration [29,30]. A sensitivity of 94% and a specificity of 83% were reported in these studies, indicating the evolving role of this diagnostic alternative for early detection of osteomyelitis, particularly in challenging cases [29,30].

It is essential to highlight that this imaging technique has no therapeutic effect on the patient; it functions solely in conjunction with technetium-99m to obtain a radioactive diagnostic agent and should not be administered directly to the patient [31]. Dosage adjustments are required for children and young individuals, applying only in cases where the benefits outweigh the risks. Before the study, discontinuation of medications containing fluoroquinolone quinolones or drugs containing calcium, sucralfate, aluminum, or magnesium is necessary, as these may alter the agent’s bioavailability. Notably, the only contraindication is hypersensitivity to the drug molecule [31].

One potential limitation of this alternative is the lack of sufficient providers to distribute the kits for diagnostic studies. Table 1 presents the characteristics of the different radioisotopes mentioned.

**Table 1:** Characteristics of the studied radioisotopes 99Mo^β^: molybdenum; Cd: cadmium 111; Zn: zinc 68

Radioisotopes	Half-life of decay	Emitting energy	Creation process	Nuclear 89 reaction
Gallium-67 (^67^Ga)	77.8 hours	Gamma rays	Cyclotron	^68^Zn(p,2n)^67^Ga
Indium-111 (^111^In)	67.2 hours	Gamma and X-rays	Cyclotron	^111^Cd(p,n)^111^In
Technetium-99m (^99m^Tc)	6 hours	Gamma rays	Nuclear reactor, Mo-99/Tc-99m generator	^99^Mo^β- ^ ---> ^99m^Tc

Three-phase bone scintigraphy: This imaging technique primarily aids in identifying bone-related infections. However, it can also detect other abnormalities, such as bone wear or fractures. The three-phase bone scintigraphy involves capturing images at specific intervals following the administration of a radiopharmaceutical. The first set of images is acquired within seconds, while the final images are typically obtained up to four hours later [32].

The three-phase bone scintigraphy consists of three phases: the first is the radionuclide angiogram, which involves the dynamic analysis of blood flow through the radiopharmaceutical. Usually, images are taken every three seconds during the first minute of radiopharmaceutical application [33,34]. The second phase is the vascular pool tracing, performed minutes after the radiopharmaceutical is applied, used to observe activity at the vascular and soft tissue levels before the tracer is absorbed by the bone tissue [34]. The third phase, bone tracing, is conducted to allow a significant amount of the radiopharmaceutical to accumulate in the tissue. In this phase, it is necessary to wait a little longer, at least about three hours, for the images to become clearer. There is a fourth phase of static imaging 24 hours after the application of the radioactive material; this last image is optional and conducted if the doctor deems it necessary [34].

**Figure 1:**
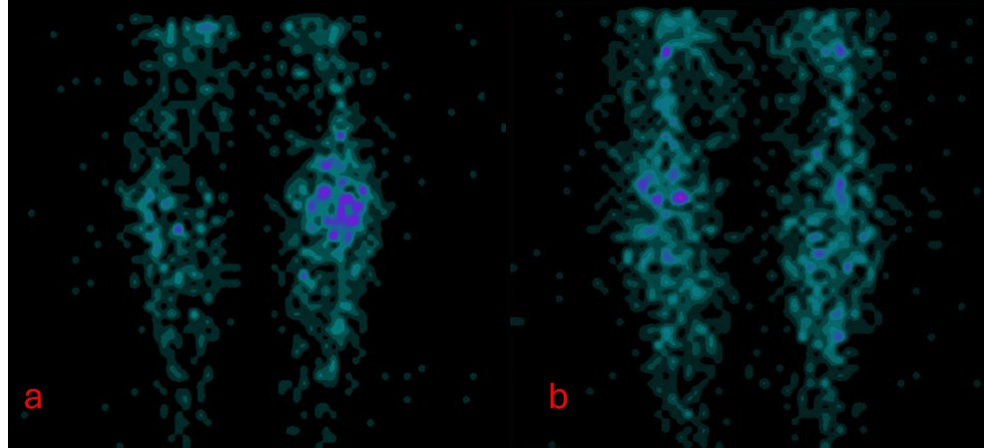
Bone scintigraphy, flow phase with 99mTc-MDP (a) Anterior view of the flow phase with 99mTc-MDP through the lower limbs. (b) Posterior view of flow phase with 99mTc-MDP through the lower limbs.

Analyzing all the information presented throughout the article and comparing the proposed alternatives for the detection of osteoarticular infections, specifically osteomyelitis, it is highlighted that the most viable option is 99mTc-ciprofloxacin for several reasons. Among them is its economic viability, as although the radioisotope has a high cost, it is lower compared to other available options. Additionally, it emits a lower amount of radiation to the patient, and its preparation and execution conditions can be carried out with the standard equipment available in a nuclear medicine pharmacy. Its use can also be more straightforward, as the preparation involves the radioisotope commonly used for preparing radiopharmaceuticals. Figure 1 presents the bone scintigraphy, angiogram phase with 99mTc-MDP.

In Figure 1, the focus is on the trajectory of the radiopharmaceutical through the bloodstream seconds after its administration to the patient. This monitoring allows for the identification of potential accumulations of the radiopharmaceutical in areas of the flow, which may suggest the presence of inflammation or infection.

**Figure 2:**
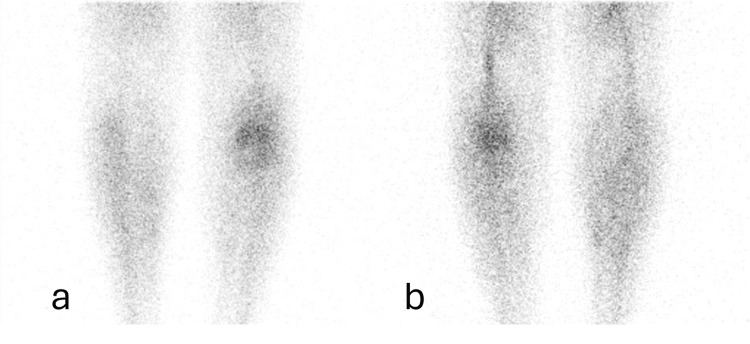
Bone scintigraphy with 99mTc-MDP, vascular pooling phase (a) Anterior view of the vascular pooling phase with 99mTc-MDP through the lower limbs. (b) Posterior view of the vascular pooling phase with 99mTc-MDP through the lower limbs.

Figure 2 shows bone scintigraphy with 99mTc-MDP, vascular pooling phase.

In Figure 2, a significant increase in vascularization of the affected region is evident during this phase, suggesting the presence of an inflammatory process in the soft tissues.

**Figure 3:**
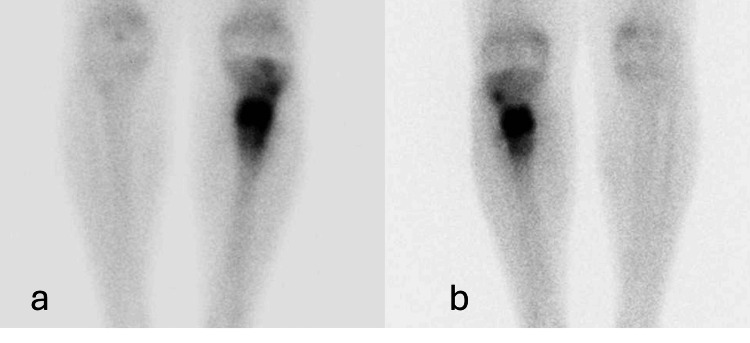
Bone scintigraphy with 99mTc-MDP, static bone tracing phase (a) Anterior view of static bone tracing phase with 99mTc-MDP in the lower limbs. (b) Posterior view of static bone tracing phase with 99mTc-MDP in the lower limbs.

Figure 3 shows bone scintigraphy with 99mTc-MDP, static bone tracing phase.

In Figure 3, in this phase, hyper-uptake is observed in the lesion area, indicating the presence of some inflammatory or infectious process.

**Figure 4:**
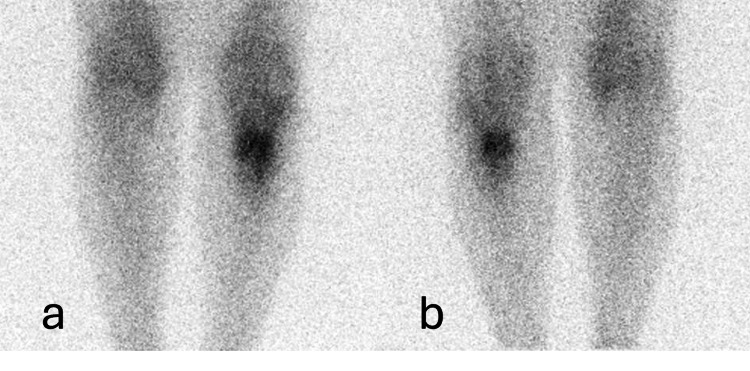
Bone scintigraphy with gallium-67 (a) Anterior view bone scintigraphy with gallium-67 in lower limbs. (b) Posterior view bone scintigraphy with gallium-67 in lower limbs.

Finally, Figure 4 shows a bone scintigraphy with 67Ga (gallium-67).

In Figure 4, the complementary scintigraphy with gallium-67 performed on the patient is observed, confirming the presence of an infectious process in the analyzed area.

Novel diagnostic tools

PET-CT and PET-MRI are emerging nuclear medicine technologies primarily used for obtaining clinical information from patients. These advanced imaging modalities are capable of detecting cancerous processes and providing detailed structural tissue information. Their high resolution has recently enabled their application as diagnostic tools for osteomyelitis [35].

Fluorine-18 fluorodeoxyglucose (18F-FDG), a widely utilized glucose analog radiotracer, is integral in PET imaging due to its reliance on the Warburg effect [36,37]. Activated leukocytes such as granulocytes and macrophages exhibit heightened glucose consumption in response to inflammation, infection, or rapid cell division, as seen in tumors. PET-CT with 18F-FDG serves as a noninvasive tool for diagnosing osteomyelitis, distinguishing between pathological fractures and insufficiency fractures, and precisely localizing infections, with a sensitivity of 94% and a specificity of up to 100%. Recent advancements have integrated 18F-FDG PET with magnetic resonance imaging, aiming to reduce radiation exposure and yield promising diagnostic outcomes [36].

Furthermore, gallium-68 pentixafor is a chemokine receptor CXCR4 ligand used in PET/CT imaging, particularly noted for its application in diagnosing chronic skeletal infections [38,39]. A recent study reported positive gallium-68 pentixafor PET/CT findings in nine out of 14 patients, demonstrating its efficacy in detecting conditions such as osteolysis, osteomyelitis, and infected prostheses. Among these positive cases, eight were confirmed through pathology, bacteriology, or clinical follow-up. Therefore, gallium-68 pentixafor PET/CT shows promise for diagnosing chronic bone infections, providing a potential diagnostic advantage in challenging cases where other imaging modalities may fall short [38].

Continued research and advancements in nuclear imaging promise further refinements and broader applications in managing complex medical conditions, reaffirming their indispensable role in modern diagnostic and therapeutic strategies [39]. These innovations not only enhance the sensitivity and specificity of imaging techniques but also facilitate personalized medicine by providing precise anatomical and functional information. Furthermore, the integration of artificial intelligence and machine learning holds the potential to revolutionize image interpretation, enabling more accurate diagnosis and treatment planning [40]. As these technologies evolve, nuclear imaging is expected to play an increasingly integral role in early disease detection, treatment monitoring, and guiding targeted interventions, thereby advancing patient outcomes and healthcare delivery on a global scale.

## Conclusions

After carefully analyzing the content of the article and considering the decrease in the production of gallium-67, a global concern arises. The potential limitations of various studies in the diagnosis of infections, especially osteomyelitis, have been highlighted. From this analysis, it is evident that the most viable alternative to gallium-67 is 99mTc-ciprofloxacin for several reasons. Primarily, its economic cost is more affordable, as, although the radioisotope carries a high price, it is lower compared to other available options. Additionally, it exposes the patient to a lower amount of radiation, and its preparation and execution can be carried out with the standard equipment available in a nuclear medicine pharmacy. It would also simplify implementation, as the preparation involves the use of the radioisotopes commonly employed in the preparation of other radiopharmaceuticals. However, further research is required to ensure and provide better alternatives for these radiopharmaceuticals.
